# Differences in EEG Event-Related Potentials during Dual Task in Parkinson’s Disease Carriers and Non-Carriers of the G2019S-LRRK2 Mutation

**DOI:** 10.3390/s23198266

**Published:** 2023-10-06

**Authors:** Eden Shkury, Shani Danziger-Schragenheim, Zoya Katzir, Yael Ezra, Nir Giladi, Anat Mirelman, Inbal Maidan

**Affiliations:** 1 Laboratory of Early Markers of Neurodegeneration, Neurological Institute, Tel Aviv Sourasky Medical Center, Tel Aviv 6423906, Israel; eden868@gmail.com (E.S.); shani1008@gmail.com (S.D.-S.); zoyakatzir@mail.tau.ac.il (Z.K.); yaelezra@mail.tau.ac.il (Y.E.); nirg@tlvmc.gov.il (N.G.); anatmi@tlvmc.gov.il (A.M.); 2 School of Medicine, Tel Aviv University, Tel Aviv 6997801, Israel; 3 Sagol School of Neuroscience, Tel Aviv University, Tel Aviv 6997801, Israel

**Keywords:** Parkinson’s disease, cognition, LRRK2, electroencephalography (EEG), event-related potential (ERP)

## Abstract

Background: The G2019S-*LRRK2* gene mutation is a common cause of hereditary Parkinson’s disease (PD), associated with a higher frequency of the postural instability gait difficulty (PIGD) motor phenotype yet with preserved cognition. This study investigated neurophysiological changes during motor and cognitive tasks in PD patients with and without the *G2019S-LRRK2* mutation. Methods: 33 iPD patients and 22 *LRRK2-PD* patients performed the visual Go/NoGo task (VGNG) during sitting (single-task) and walking (dual-task) while wearing a 64-channel EEG cap. Event-related potentials (ERP) from Fz and Pz, specifically N200 and P300, were extracted and analyzed to quantify brain activity patterns. Results: The *LRRK2-PD* group performed better in the VGNG than the iPD group (group*task; *p* = 0.05). During Go, the iPD group showed reduced N2 amplitude and prolonged N2 latency during walking, whereas the *LRRK2-PD* group showed only shorter latency (group*task *p* = 0.027). During NoGo, opposite patterns emerged; the iPD group showed reduced N2 and increased P3 amplitudes during walking while the *LRRK2-PD* group demonstrated increased N2 and reduced P3 (N2: group*task, *p* = 0.010, P3: group*task, *p* = 0.012). Conclusions: The *LRRK2-PD* group showed efficient early cognitive processes, reflected by N2, resulting in greater neural synchronization and prominent ERPs. These processes are possibly the underlying mechanisms for the observed better cognitive performance as compared to the iPD group. As such, future applications of intelligent medical sensing should be capable of capturing these electrophysiological patterns in order to enhance motor–cognitive functions.

## 1. Introduction

In recent decades, a substantial body of evidence has shown the involvement of genetics in Parkinson’s disease (PD) development. Numerous associated genes and susceptibility loci have been identified [1]. One significant gene is the leucine-rich repeat kinase 2 (*LRRK2*) gene. Mutations in *LRRK2* are a common cause of hereditary PD [2]. Among the various *LRRK2* mutations, the *G2019S* change in exon 41 is the most frequent and extensively studied. This mutation is particularly noticeable in Ashkenazi Jewish patients, with a carrier frequency of 29.7% among familial cases and 13.3% in sporadic PD cases [2,3]. The clinical phenotype of *LRRK2* mutation carriers is often perceived as similar to that of idiopathic PD (iPD) patients. However, research has revealed that carriers tend to experience a relatively benign disease course, expressed with increased rigidity and balance disturbances, known as the postural instability gait deficit (PIGD) motor phenotype. In terms of treatment, *LRRK2* carriers have shown a favorable response to L-dopa therapy [4,5,6]. Although the PIGD phenotype in iPD patients is associated with worse cognitive performance, *LRRK2* mutation carriers demonstrate relatively preserved cognitive function with better attention, executive functions, and language skills compared to non-carriers [7,8]. However, no differences were observed in the MoCA test scores or in memory, visuospatial, and psychomotor speed tasks [8]. A number of functional MRI studies provide some evidence that can explain the preserved cognitive abilities in *LRRK-PD*. For example, decreased functional connectivity between the putamen and the bilateral superior frontal gyri, precuneus, and calcarine gyri have been noted in *LRRK-PD* compared to iPD [9]. Additionally, a greater loss of neuromelanin, which reflects neuronal death, in the locus coeruleus and red nucleus was found in iPD compared to *LRRK-PD* [10]. However, these studies did not examine ongoing cognitive processes that directly represent cognitive function.

Electroencephalography (EEG) is a direct measurement of neuronal electrical activity that is being used to analyze cognitive function in different neurological diseases such as PD [10,11]. Event-related potentials (ERPs) extracted from the EEG recordings reflect the perception and processing of both sensory information as well as types of higher-level processing such as selective attention, memory updating, and semantic comprehension. Different ERPs have been attributed to these different levels of cognitive processing, and alterations in their amplitude and latency have been used to measure different aspects of electrical brain activity [10]. Previous studies have shown differences in ERP components between PD patients and healthy controls when performing cognitive tasks, such as the visual Go/NoGo task (VGNG), relating to the severity of the patient’s cognitive dysfunction [12,13]. The ERP changes were mainly observed in P300, an index of stimulus processing and the most studied ERP related to attention and cognitive decline [14,15]; changes in P300 were found to be highly sensitive to cognitive deterioration and attentional impairments [12]. More specifically, P300 amplitude represents the sum of all the extracellular currents that are time-locked to a task; therefore, it has been related to the quantity of attention resources engaged during the task [16,17,18]. Patients with PD have been reported to have decreased P300 amplitude compared to healthy older adults as the severity of cognitive impairment increases [17]. Another component of P300 is its latency, which had been shown to reflect the relative time to evaluate different stimuli including the processes necessary for making a task-relevant decision [19]. P300 latency was prolonged in PD patients when compared to healthy controls [20]. In a recent comprehensive meta-analysis focusing on N200 and P300 components in PD patients, it was observed that the P300 effect was predominantly evident at the Fz site, with no significant variations detected at other electrode sites [21]. Intriguingly, the analysis also revealed that the P300 latency in PD patients diagnosed with dementia (PDD) was notably prolonged when compared to their non-dementia PD counterparts (PDND). This compelling finding led to the inference that as attention and working memory deteriorate in PD, the latency of P300 increases, potentially signifying an early dysfunction in the processing of cognitive information and attention. Another recent study revealed similar differences in P300 amplitude between PD patients with minimal cognitive impairment (MCI) and cognitive–normal (CN) PD patients, further supporting the notion that P300 amplitude may serve as a valuable marker for identifying early cognitive impairments related to attention and information processing in PD [21,22]. Similar differences were demonstrated in an additional study that specifically examined early-stage drug-naive (ESDN) PD patients. This study found that ESDN PD patients exhibited both decreased P300 amplitude and prolonged latency when compared to healthy controls. These findings suggest that even in the early stages of PD, before any exposure to medication, cognitive deficits are already evident among these patients and can be detected using EEG [23].

The N200 is an ERP typically following the presentation of a specific visual or auditory stimulus; it is typically evoked before the motor response, suggesting its link to the cognitive processes of stimulus identification and distinction [24]. Past research focused on the N200 as a mismatch detector, but it has also been found to reflect executive cognitive control functions [25]. More generally, the N200 component has been described in tasks that reflect stimulus identification, attentional shifts, the inhibition of motor responses, overcoming stereotypical responses or conflict monitoring, the maintenance of context information, response selection timing, and the detection of novelty or mismatch [20,24,25,26,27]. N200 amplitude had shown to be decreased in PD patients in comparison to healthy controls [21,28,29,30]. These findings may suggest that in initial stages of stimulus processing, PD patients could be impaired in mismatch detection and the subsequent classification of a presented stimulus as standard, target, and distracter [29]. The N200 latency showed a significant prolongation when comparing PD patients to healthy controls, indicating that the N200 component may serve as an indicator of early cognitive impairment in PD patients [21]. In another recent study using the oddball paradigm, the N200 component elicited from control subjects had a shorter latency and greater amplitude compared to that from PD patients. These differences may reflect deficits in auditory processing or executive functions in the PD group, perhaps being early indicators of impending cognitive decline in some of these patients [30]. Altogether, the EEG of PD patients showed typical morphology of P300 that included decreased amplitude along with longer latency. Changes in N2 included only prolonged latency. These findings correlated with a delay in the cognitive processing of information, a decrease in the intensity of selective attention processes, and asynchrony in neural activation in PD patients [31]. Furthermore, recent studies have used EEG recordings to differentiate between PD patients and healthy controls, highlighting EEG’s potential as a reliable, cost-effective, and readily available tool for PD detection and disease progression monitoring [32,33,34].

PD involves deficits in motor and cognitive functions that are often exacerbated during dual tasks in which motor and cognitive tasks are preformed simultaneously [35]. It has been theorized that walking in everyday life conditions requires higher cognitive processes while handling the competing demands from the environment is treated as maintaining motor performance [36]. Engaging in additional attention-demanding tasks while walking enforces the recruitment of cortical motor and cognitive resources. When those compensatory mechanisms are overwhelmed or impaired, or when resources are limited, task performance will be impacted; in this example, either the cognitive task gait or both will be impacted. A common way to investigate the interaction between motor and cognitive functions is by using a dual-task walking paradigm [37]. Previous studies have considered the interaction between gait and visual and cognitive functions and emphasized the crucial role of visual attention during gait as a means for selectively tending to specific stimuli while suppressing others. The “Go/NoGo” task has been widely used to measure participants’ capacity for this kind of sustained attention and response control as it requires overcoming the potent response tendency, generated by frequent ‘Go’ stimuli, to successfully inhibit the execution of responses to ‘NoGo’ stimuli [38].

Previous studies have shown that response inhibition during walking is impaired in PD patients and that increase cognitive load during dual-task walking relates to significant changes in scalp electrical activity, mainly in the parietal and fronto-central channels [39]. ERP changes during dual-task walking have been observed in older adults and PD patients; however, no previous study has explored whether these mechanisms vary between different motor and cognitive phenotypes of PD [20]. The combination of genetic factors related to specific motor–cognitive phenotypes and electrophysiological patterns revealing brain activity may aid in the development of an intelligent sensor [40] to maximize the potential of motor–cognitive function in PD patients. Therefore, in this study, we aimed to examine the variations in ERPs during dual task performance across PD patients with different motor and cognitive phenotypes, specifically comparing *LRRK2-PD* patients, who show better cognitive function, to idiopathic PD (iPD) patients. We hypothesized that *LRRK2-PD* patients would demonstrate increased ERP amplitudes during dual tasks compared to iPD patients, which would be related to better performance on the cognitive task, potentially serving as a future intelligent biomarker.

## 2. Materials and Methods

### 2.1. Participants

The study included 33 iPD patients and 22 *LRRK2-PD* patients. PD patients were eligible for inclusion if they had been diagnosed within the past five years and had Hohen & Yahr (HY) scores of ≤ 2. Participants were excluded if they had severe cognitive decline (Montreal Cognitive Assessment (MoCA) score below 21) [41], a history of head trauma or neurologic disorders, unstable medical conditions including cardio-vascular instability, or significant psychiatric co-morbidity. The study was approved by the local ethical committee and was performed according to the principles of the Declaration of Helsinki. All participants gave their informed written consent prior to participation.

### 2.2. Study Protocol

All eligible participants underwent a clinical evaluation and an EEG recording. The EEG examination involved performing the vGNG task both while seated (single task, ST) and while walking on a treadmill (dual task, DT). The treadmill walking speed was adjusted to each subject’s comfort level, and they were instructed to keep their hands on the treadmill rail during the test to minimize movement artifacts. In the VGNG task, the participants were presented with two types of visual stimuli randomly. They were instructed to respond overtly (e.g., press a button) when they saw an English letter (the “Go” cues) and to withhold their response (not press the button) when the letter X was presented (the “NoGo” cues). Each task consisted of two sessions, each lasting six minutes, with a one-minute break in between (a total of 12 min of sitting and 12 min of walking). The ratio of frequent ‘Go’ to ‘NoGo’ events was 4:1 (or 80% vs. 20%), resulting in a total of 400 events. Following the EEG recording, participants underwent cognitive, gait, and clinical assessments. The cognitive assessment comprised the Montreal Cognitive Assessment (MoCA) [42] and the color trail test (CTT), used to evaluate visual scanning, attention, inhibition, and cognitive flexibility [43]. The gait assessment included walking overground in a 10 m corridor, during which the patients walked back and forth for a minute, once at their comfortable speed (usual walking) and once while subtracting 7 from a 3-digit number (dual-task walking). Gait performance was measured via gait speed (meters per second). The clinical assessment covered demographic information and the Movement Disorders Society Unified Parkinson’s Disease Rating Scale (MDS-UPDRS) assessed PD symptom severity [44]. In addition,, the patients’ medication intake levels were recorded in terms of L-dopa equivalent daily dose (LEDD) [45].

### 2.3. EEG Acquisition and Preprocessing

A 64-channel EEG system (EGI GES400) recorded electrical brain activation at 250 Hz sampling rate. Out of the 64 electrodes, four were used to measuring eye movement, with two placed above and below the right eye for vertical EOG and the other two positioned at the outer canthuses of both eyes for horizontal EOG. Electrode position was set according to the International 10-10 standard. EEG data were band-pass-filtered with a zero-phase-lag Finite Impulse Response (FIR) filter (0.5–40 Hz). Independent Component Analysis (ICA) was applied for blink artifact removal, and components related to blinks were subtracted from the data. The signal was divided into 1.25 s epochs of −250 msec pre-event and 1000 msec post-event. Epochs with reaction times shorter than 150 msec and greater than 4 SD from the mean were removed. The amplitude and latency of the N2 and P3 components were extracted from channels Fz and Pz for ERP analysis. We selected, specifically, the Fz and Pz electrodes, since the N2 component is usually larger in the anterior and frontocentral scalp sites [10,14] and P3 component is mainly produced by parietal and inferior temporal areas [10,14].

### 2.4. Statistical Analysis

The means and standard deviations of all the demographic and cognitive variables were calculated and evaluated for normality and homogeneity of variance using Q-Q plot and Levene’s homogeneity test, respectively. Independent *t*-test was used to examine differences between iPD and *LRRK2-PD* groups in demographics and behavioral measures. Differences in gender were examined using Chi-square test. Linear mixed models were used to examine the effects of group (iPD and *LRRK2-PD*), task (sit, walk), and their interactions on measures of VGNG performance (correct % and response time) and measures of ERPs (N2 and P3 amplitude and latency). ERPs of Go cues and NoGo cues in Fz and Pz were examined separately. Pearson’s correlations between behavioral measures (correct % and response time) and ERP measures (N2 and P3 amplitudes and latencies) were examined in all subjects together. The significance level was set to *p* = 0.05 and corrected for multiple comparisons. The statistical analyses were performed using SPSS software version 29.

## 3. Results

### 3.1. Participants

As shown in Table 1, iPD patients demonstrated longer times to complete CTT 1 and 2 (*p* = 0.003, *p* = 0.014) and higher scores in total MDS-UPDRS (*p* = 0.043) and MDS-UPDRS part III (*p* = 0.007). No significant differences were observed in age, gender, years of education, MOCA, and gait speed.

### 3.2. Visual Go/NoGo Performance

**Figure 1:**
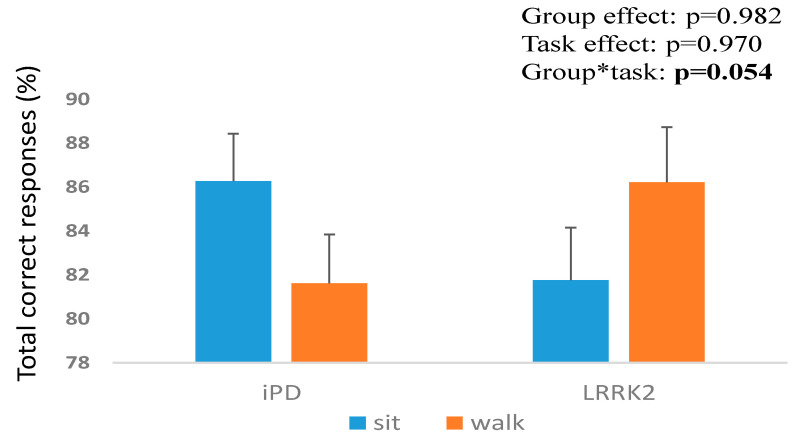
Differences in total correct percent in the VGNG task during sitting and walking between iPD and *LRRK2-PD* groups. * significant group*task interaction.

The iPD group demonstrated a lower percent of total correct responses (worse performance) during walking compared to sitting. In contrast, the *LRRK2-PD* group showed a higher percent of total correct responses (better performance) during walking compared to sitting (group*task interaction, *p* = 0.054) (Figure 1). Both groups showed reduced NoGo correct percent during walking compared to sitting (task effect; *p* = 0.010) with no group effect (*p* = 0.483). No significant difference in the Go response time was found between the groups (*p* = 0.891) tasks (*p* = 0.124) and task x group interaction (*p* = 0.227).

### 3.3. Event-Related Potentials

#### 3.3.1. Go cues

**Figure 2:**
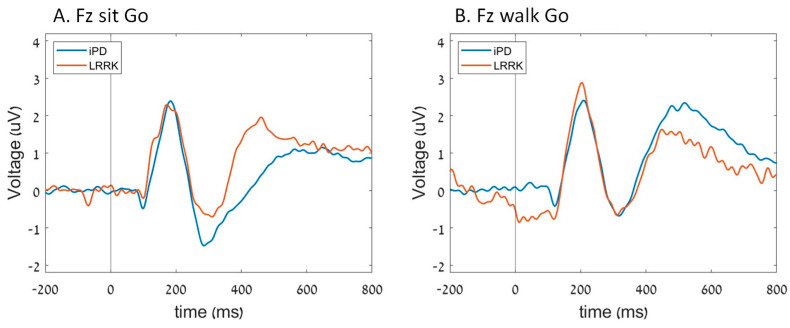
The average ERP of each group in Fz electrode during (**A**) sit Go cues and (**B**) walk Go cues.

**Figure 3:**
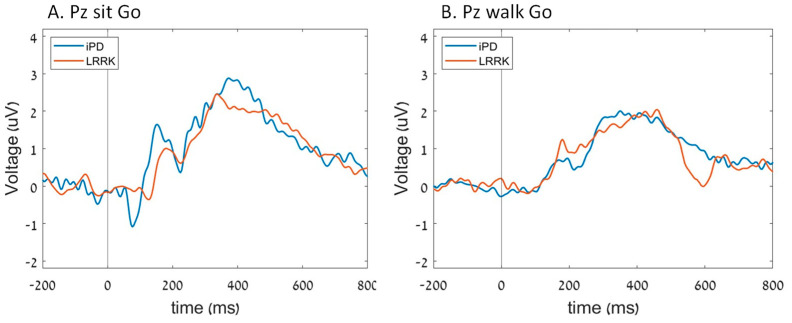
The average ERP of each group in Pz electrode during (**A**) sit Go cues and (**B**) walk Go cues.

In the iPD group, the N2 amplitude in Fz was less prominent (closer to zero) during walking compared to sitting. However, in the *LRRK2* group, no significant differences were found in N2 amplitude between sitting and walking (group*task interaction; *p* = 0.027) (Figure 2). Furthermore, there were no significant differences in N2 amplitude in Pz between the groups (*p* = 0.118), tasks (*p* = 0.202), and their interaction (*p* = 0.429) (Figure 3). The P300 amplitudes were similar for the groups (*p* = 0.958; *p* = 0.402), tasks (*p* = 0.498; *p* = 0.204), and their interaction (*p* = 0.250; *p* = 0.117) in both Pz and Fz.

Different patterns in N2 latency in Fz were found between the groups during sitting and walking (group*task interaction; *p* = 0.019). While the iPD group showed longer N2 latency during walking compared to sitting, the LRRK2-PD group demonstrated shorter latency during walking compared to sitting (Figure 2). N2 latency values in Pz were not different for the groups (*p* = 0.657), tasks (*p* = 0.341), and their interaction (*p* = 0.111) (Figure 3), with no differences in P300 latency between the groups (*p* = 0.212; *p* = 0.109), tasks (*p* = 0.296; *p* = 0.101), and their interaction (*p* = 0.429; *p* = 0.723) in either Pz or Fz.

#### 3.3.2. NoGo cues

**Figure 4:**
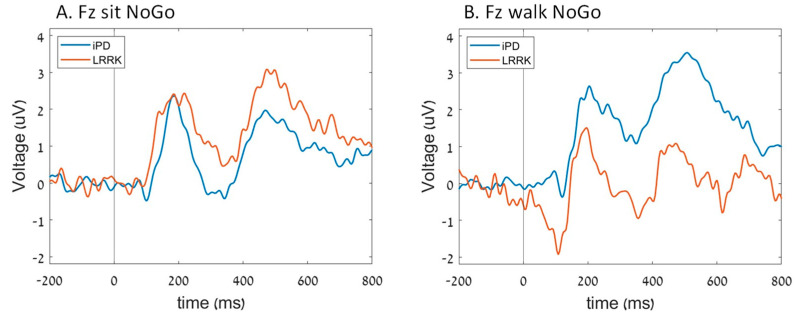
The average ERP of each group in Fz electrode during (**A**) sit NoGo cues and (**B**) walk NoGo cues.

In the iPD group, N2 amplitude in Fz was less prominent during walking compared to sitting, whereas for the *LRRK2-PD*, the results were the opposite, showing more prominent N2 amplitude while walking (group*task interaction; *p* = 0.010) (Figure 4). However, no significant difference in N2 latency was observed between the groups, even though both groups exhibited similar decreases in latency while performing the dual task (*p* = 0.025). Moreover, there was no significant interaction between task and group (*p* = 0.965).

**Figure 5:**
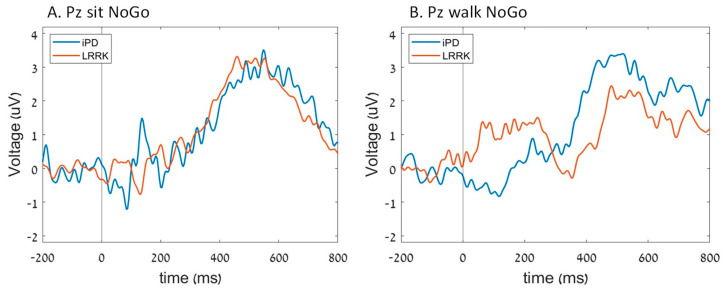
The average ERP of each group in Pz electrode during (**A**) sit NoGo cues and (**B**) walk NoGo cues.

For the Fz P300 amplitude, we observed that in the iPD group, the P300 amplitude increased during walking compared to sitting, whereas in the *LRRK2-PD* group, it was the opposite, with the P300 amplitude decreasing during walking compared to sitting (group*task interaction; *p* = 0.012) (Figure 4). However, no significant differences in Fz P300 latency were observed between the groups. At Pz, no differences were found between the groups in either the amplitude or latency of N2 and P300 (Figure 5).

All ERPs results are summarized in Table 2 below. In addition, the mean amplitudes and latencies of all Fz ERPs are presented in Supplementary Tables S1 and S2, and scalp maps showing the distribution of activations on the scalp during sitting and walking are presented in Supplementary Figure S1.

No significant correlations between measures of ERPs (N2 and P300 amplitude and latency) and measures of VGNG performance (e.g., correct responses, response time) were found.

## 4. Discussion

Visual attention and the ability to refrain from unwanted responses are important cognitive abilities required to successfully ambulate in everyday life environments. In this work, we aimed to explore the underlying neural mechanisms that may explain more preserved cognitive abilities in *LRRK2-PD* patients compared to iPD patients. Our main findings demonstrated: (1) significant differences between the groups only in the Fz channel; (2) opposite changes in N2 amplitude and latency between the groups in differences between single- and dual-task patters, wherein the iPD group showed a decreased N200 amplitude and prolonged latency while the *LRRK2-PD* group showed an increase in N2 amplitude and shorter latency during walking compared to sitting; (3) opposite patterns in terms of P300 amplitude between the groups, with the iPD group showing an increase in P300 amplitude and the *LRRK2-PD* group showing a decrease in P300 amplitude during walking compared to sitting; and (4) the *LRRK2-PD* group showing a difference between tasks requiring response inhibition. There was an increase in N200 amplitude during walking in the NoGo events, but not in the Go events, and a prolonged N2 latency during NoGo events, which was shortened during Go events. Recent advances in technology, particularly in artificial intelligence analysis, may pave the way for the development of intelligent sensors aligned with the current trends in personalized medicine and health monitoring [40].

Visual attention is modulated by various regions in the brain including the basal ganglia–frontal neuronal network [46]. The frontal cortex, specifically the prefrontal cortex (PFC) and its related circuits, plays a crucial rule in planning, organization, execution, and adjustment to the environment while walking [47,48]. Alterations in the networks that involve the basal ganalia–frontal areas may impair the ability to ignore distracting stimuli and result in poor response selection [46,49,50,51]. Several studies that used functional near-infrared spectroscopy (fNIRS) to examine frontal lobe activity during walking provided direct evidence for frontal activity during dual-task walking [52,53,54]. Although deficits in executive function are common among patients with PD [55,56,57], recent evidence suggests that patients utilize the frontal lobe, mainly regions associated with cognitive resources, to compensate for cognitive impairments associated with gait [58,59,60]. The fact that all the ERPs changes found in this study were in the Fz channel may suggest that cognitive differences rely on changes in more frontal cognition-oriented areas. This is a finding that stands in line with previous studies and highlights the important compensatory role of the frontal lobe in PD [54].

The N200 is an ERP linked to the early cognitive processes of stimulus identification and distinction and reflects executive control functions, attentional shifts, the inhibition of motor responses, and the detection of mismatch [24,25,26,27]. Our findings revealed specific changes in the N200 patterns of Go and NoGo cues during walking in *LRRK2-PD* and iPD patients. The iPD patients demonstrated a decreased N2 amplitude during walking compared to sitting for both Go and NoGo cues while the *LRRK2-PD* patients showed no changes in amplitude for the Go cues but an increased N2 amplitude for the NoGo cues during walking. An increase in N200 amplitude is linked to the activation of neuronal networks and higher neuronal synchrony [24]. Thus, the observed changes in N2 amplitude for NoGo cues during walking may suggest that the better cognitive performance in *LRRK-PD* patients is related to enhanced synchronization and more efficient compensatory mechanisms, especially during the initial cognitive processing of NoGo cues while walking—a complex task that requires the involvement of multiple brain resources [25,26,27].

In terms of the N2 latency of Go cues, the iPD group demonstrated prolonged latency whereas the *LRRK2-PD* patients showed a shortening of latency during walking compared to sitting. For the NoGo cues, both groups presented shorter latency during walking. The latency component has been related to cognitive processing speed wherein longer latency indicates slower cognitive processing. Thus, the shorter latency observed in the *LRRK2-PD* group during both Go and NoGo cues while walking suggests potential higher arousal or an overlap between motor and cognitive networks during the initial cognitive processes while walking. In contrast, in the iPD patients, a longer latency during walking was observed for the Go cues, suggesting specific slower initial cognitive processing in attention. However, these effects did not impact later cognitive processes and motor execution, as indicated by no differences in response time and total correct responses in each condition separately.

The P300 is an index of stimuli processing that has been considered a motor-free measure of cognitive function [14,15]. Its amplitude is a sum of all the extracellular currents that are time-locked to a task. Therefore, it has been related to the number of synchronized neurons that are activated during the processing of incoming information [16,17]. The lack of differences in P300 amplitude during Go cues between sitting and walking in both groups suggests that the recruitment of attentional resources during this relatively simple task is preserved in both groups. However, for NoGo cues, which involve more complex responses, the increased P300 amplitude during dual tasking in the iPD group and the decreased P300 amplitude in the *LRRK2-PD* group may reflect alterations in later cognitive control processes between the two groups [44]. This increase in P300 amplitude in the more complex NoGo task and while dual tasking in the iPD patients may reflect an inefficient compensation. In contrast, the decrease in P300 amplitude observed in the *LRRK-PD* group during walking may suggest that additional brain resources were recruited for later cognitive processes to successfully perform the task, impacting the P300 amplitude. The lack of significant correlations between measures of ERPs (N2 and P300 amplitude and latency) and measures of VGNG performance could potentially be attributed to the small differences in task performance between the PD groups, given the relatively limited sample size.

We acknowledge several limitations in our study. First, the sample size was relatively small, and a larger sample may yield more significant results. Nevertheless, conducting research involving unique populations like LRRK-2 carriers inherently limits the pool of potential participants. This approach aligns with the established practices observed in numerous EEG studies across diverse fields of research [61,62,63]. These studies collectively validate the judicious use of smaller participant groups, particularly in specialized research domains. By focusing on LRRK-2 carriers, our study maintained consistency with this methodological approach, ensuring the relevance and accuracy of our findings. Second, it is important to note the absence of a healthy control group for comparison. Nevertheless, our group has published several studies comparing PD patients with healthy individuals [20,39]. These studies consistently demonstrated that, during dual task conditions, PD patients exhibit a lower and delayed P300 amplitude compared to controls. In this study, our primary objective was to investigate specific differences between iPD and LRRK2-PD, especially given the relatively intact cognition in LRRK2-PD patients. In addition, the selection of this design for our study has been substantiated by recent research in the EEG domain [64,65,66]. These studies collectively highlight the significance of considering both task-driven and subject-driven EEG signal variations. EEG signals can exhibit variability due to the nature of tasks and the unique characteristics of individual subjects. By employing this design, we were poised to capture and differentiate these nuanced fluctuations within our participant group.

Last, our findings reflect the electrical changes observed on the scalp, which represent the sum of multiple brain generator activations. As a result, these findings cannot be attributed to specific brain areas although all our observations were made at the Fz electrode and not the Pz electrode. Future studies should incorporate tools for source localization analysis, such as the sLORETA, to gain a better understanding of the brain generators involved. Additionally, they must consider incorporating event-related synchronization and desynchronization (ERS and ERD) components to investigate alterations in the frequency domain. It is worth noting that the walking was performed on a treadmill while holding the rails. Overground walking differs from treadmill walking, especially in PD patients, and this distinction should be taken into consideration in future studies. For example, studies may involve performing overground walking for one minute, both with and without a Go/NoGo task, during which the subjects walk back and forth in a 20 m corridor. Examining our paradigm during overground walking presents challenges due to the significant noise contamination of the EEG signal, but it is crucial for understanding real-world implications. In addition, future studies should include various types of dual-task paradigms that could have different effects.

## 5. Conclusions

This study aimed to explore the underlying neural mechanisms that might explain the superior cognitive abilities of *LRRK2-PD* patients compared to iPD patients, utilizing EEG signals and ERP waveforms. We successfully identified differences between the groups associated with dual tasking, providing direct evidence of distinct differences in electrical brain activity in the *LRRK2-PD* group compared to the iPD group. Our results indicate that *LRRK2-PD* patients utilize early cognitive resources potentially as compensatory mechanisms for more complex tasks, likely relying on better synchronization compared to iPD patients. Furthermore, the findings of this study shed light on the differences in neural activation during cognitive processing between *LRRK2-PD* and iPD patients, partially explaining preserved cognitive performance. These insights may pave the way for the development of targeted multimodal interventions aimed at harnessing and enhancing the compensatory mechanisms identified in LRRK2-PD patients, which, in turn, could enhance complex tasks like dual tasking in daily life. In conclusion, this article brings together two important fields that have gained significance in Parkinson’s disease research over the last decade. The first pertains to the influential role of genetics in delineating various PD subgroups. The second involves using electrophysiology to reveal potential neurophysiological mechanisms that are pertinent for understanding the nature of the disease, especially in the context of deep brain stimulation therapy. For the first time, this article integrates the fields of genetics and electrophysiology in explaining Parkinson’s disease, unveiling the potential for developing intelligent sensing biomarkers in this domain.

## Figures and Tables

**Table 1:** Study’s population demographic characteristics.

Mean (STD)	Idiopathic PD	*LRRK2 PD*	*p*-Value
Gender (F)	F 30.3%	F 40.9%	0.422
Age (years)	67.69 (9.693)	65.57 (9.047)	0.278
Years of education	16.28 (3.314)	16.19 (2.909)	0.949
Disease duration (months)	15.95 (18.6)	17.77 (18.01)	0.843
MOCA score	25.97 (2.279)	26.21 (2.529)	0.663
Sitting serial 7 correct answers	13.82 (6.891)	20.5 (12.057)	0.101
Dual task serial 7 correct answers	12.78 (5.846)	16.2 (10.390)	0.457
CTT-1 (s)	66.47 (29.806)	44.63 (21.565)	0.003
CTT-2 (s)	122.65 (59.882)	85.85 (33.660)	0.014
MDS-UPDRS (total)	45.22 (20.632)	32.45 (16.754)	0.043
MDS-UPDRS part III (motor)	27.50 (11.706)	18.05 (10.590)	0.007
LEDD (mg)	411.77 (398.922)	307.95 (297.309)	0.321
Gait speed (m/s)	0.951 (0.210)	0.909 (0.216)	0.765
Dual task gait speed (m/s)	0.872 (0.231)	0.973 (0.420)	0.190

F = female, MOCA =Montreal Cognitive Assessment, CTT = Color Trails Test, UPDRS = Unified Parkinson’s Disease Rating Scale, LEDD = Levodopa equivalent daily dose.

**Table 2:** Summary of all ERP changes from sitting to walking.

	Idiopathic PD	LRRK2-PD
Go N200 amplitude	↓	↔
Go N200 latency	↑	↓
Go P300 amplitude	↔	↔
Go P300 latency	↔	↔
NoGo N200 amplitude	↓	↑
NoGo N200 latency	↓	↓
NoGo P300 amplitude	↑	↓
NoGo P300 latency	↔	↔

↓ reduced during walking compared to sitting; ↑ increased during walking compared to sitting; ↔ no changes between sitting and walking.

## Data Availability

Data will be shared upon any reasonable request.

## Supplementary Materials

The following supporting information can be downloaded at: https://www.mdpi.com/article/10.3390/s23198266/s1, Table S1: Summary of N2 amplitudes and latencies in Fz; Table S2: Summary of P3 amplitudes and latencies in Fz. Supplementary Figure S1: Scalp maps depicting the activation during sitting and walking in (A) LRRK-PD patients and (B) iPD patients in the time window spanning 0-650 milliseconds after the event.
