# Supplementary material for: Differences in EEG Event-Related Potentials during Dual Task in Parkinson’s Disease Carriers and Non-Carriers of the G2019S-LRRK2 Mutation
Source: Sensors (Basel). 2023 Oct 6;23(19):8266. doi: 10.3390/s23198266 (PMC10575245; doi:10.3390/s23198266)
Supplement: Supplementary file 1 [file sensors-23-08266-s001.zip › sensors-2603089-supplementary.pdf]

sensors

MDPI

Supplementary

Supplementary Table S1. Summary of N2 amplitudes and latencies in Fz.

Group	ERP Measure	Sitting	Walking
IPD	N2 amplitude Go	-2.592±0.379	-1.548±0.387
	N2 latency Go	274.9±11.4	296.4±11.8
	N2 amplitude NoGo	-2.221±0.629	-0.925±0.655
	N2 latency NoGo	272.3±14.4	246.3±14.9
LRRK2	N2 amplitude Go	-1.620±0.458	-1.617±0.464
	N2 latency Go	287.0±13.8	257.9±14.1
	N2 amplitude NoGo	-0.754±0.759	-2.789±0.780
	N2 latency NoGo	265.74±17.51	240.65±17.8

Supplementary Table S2. Summary of P3 amplitudes and latencies in Fz.

Group	ERP Measure	Sitting	Walking
IPD	P3 amplitude Go	2.174±0.727	3.585±0.759
	P3 latency Go	544.8±16.1	515.2±16.8
	P3 amplitude NoGo	3.284±0.815	5.387±0.848
	P3 latency NoGo	522.3±15.2	507.9±15.8
LRRK2	P3 amplitude Go	3.017±0.877	2.648±0.903
	P3 latency Go	506.2±19.4	502.0±20.0
	P3 amplitude NoGo	4.247±0.948	2.415±1.010
	P3 latency NoGo	476.6±18.8	508.4±18.8

Supplementary Figure S1

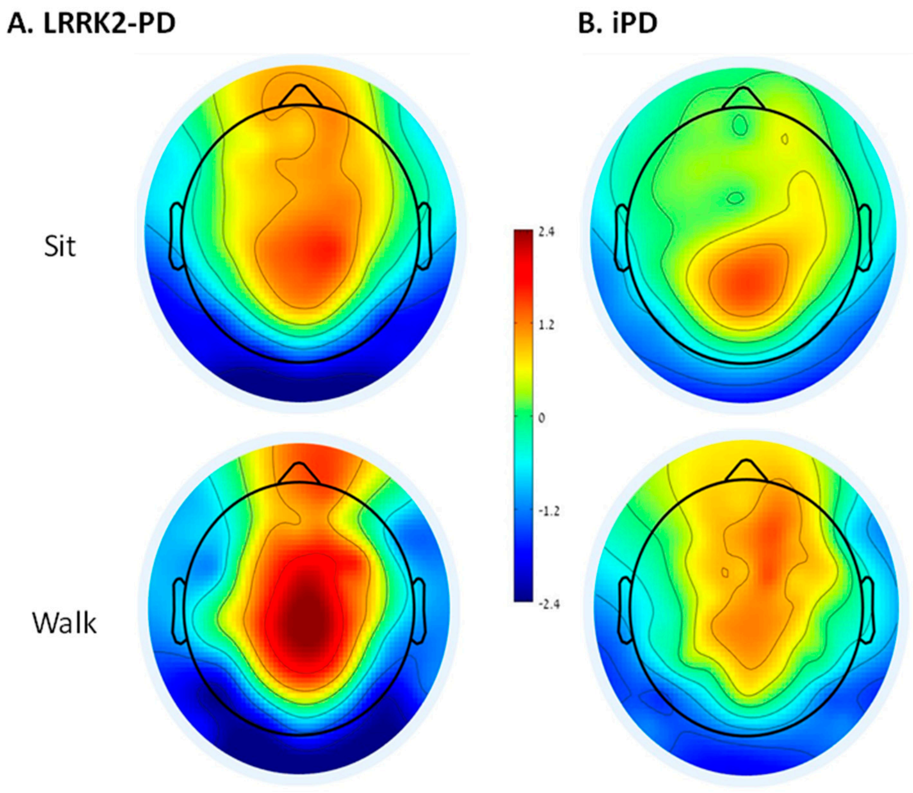

Supplementary Figure S1. Scalp maps depicting the activation during sitting and walking in (A) LRRK-PD patients and (B) iPD patients in the time window spanning 0-650 milliseconds after the event.
